# Antimicrobial Susceptibility Profiles of Commensal *Enterococcus* spp. Isolates from Turkeys in Hungarian Poultry Farms Between 2022 and 2023

**DOI:** 10.3390/antibiotics14040331

**Published:** 2025-03-21

**Authors:** Ádám Kerek, Ábel Szabó, Franciska Barnácz, Bence Csirmaz, László Kovács, Ákos Jerzsele

**Affiliations:** 1 Department of Pharmacology and Toxicology, University of Veterinary Medicine, István utca 2, H-1078 Budapest, Hungary; szabo.abel@student.univet.hu (Á.S.); barnacz.franciska@student.univet.hu (F.B.); csirmaz.bence@student.univet.hu (B.C.); jerzsele.akos@univet.hu (Á.J.); 2 National Laboratory of Infectious Animal Diseases, Antimicrobial Resistance, Veterinary Public Health and Food Chain Safety, University of Veterinary Medicine, István utca 2, H-1078 Budapest, Hungary; kovacs.laszlo@univet.hu; 3 Department of Animal Hygiene, Herd Health and Mobile Clinic, University of Veterinary Medicine, István utca 2, H-1078 Budapest, Hungary; 4 Poultry-Care Kft., Lehel út 21, H-5052 Újszász, Hungary

**Keywords:** *Enterococcus*, antimicrobial resistance, AMR, minimum inhibitory concentration, MIC, poultry, turkeys, Hungary

## Abstract

**Background:** Antimicrobial resistance (AMR) has become a serious global challenge in the 21st century. Poultry, including turkeys, are a vital source of animal-derived protein worldwide. Commensal bacterial strains in poultry can act as reservoirs for AMR, making monitoring them crucial for both veterinary and public health. *Enterococcus* species are emerging pathogens, particularly in severe nosocomial infections. **Methods:** This study aimed to assess the resistance profiles of commensal *Enterococcus* strains isolated (*n* = 470) from large-scale turkey flocks in Hungary. From each animal, two swab samples were collected: one from the oropharyngeal region near the tracheal entrance and one from the cloaca. The samples were subsequently processed, and the minimum inhibitory concentration (MIC) was determined following the Clinical and Laboratory Standards Institute (CLSI) guidelines. The tested antibiotics included amoxicillin, amoxicillin–clavulanic acid, imipenem, neomycin, doxycycline, florfenicol, tylosin, enrofloxacin, potentiated sulfonamide, vancomycin, ceftriaxone, spectinomycin, tiamulin, lincomycin, and colistin. The dilution range for MIC determination was set between 512 and 0.001 µg/mL. **Results:** Resistance to amoxicillin, a first-line treatment for *Enterococcus* infections, was low (11.1%). However, high resistance levels were observed for tylosin (62.6%), florfenicol (51.1%), doxycycline (48.7%), and enrofloxacin (45.5%). Notably, vancomycin resistance reached 15.5%, a finding consistent with global trends. Compared to human-derived *Enterococcus* data, resistance to aminopenicillins was significantly lower in turkey isolates, while neomycin resistance levels were comparable to those observed in human *E. faecalis* strains. **Conclusions:** The findings underscore the necessity of continuous surveillance of AMR trends in poultry production. While amoxicillin remains an effective treatment, the presence of multidrug-resistant strains and vancomycin-resistant isolates raises concerns regarding the potential dissemination of resistance genes. Future studies should incorporate next-generation sequencing to elucidate the genetic mechanisms underlying resistance. Additionally, integrating antibiotic usage data from farms may provide further insights into resistance dynamics. Strengthening antibiotic stewardship programs and fostering collaboration between veterinary and human medicine are crucial steps in addressing AMR under the One Health framework.

## 1. Introduction

In 2008, The European Food Safety Authority (EFSA) established an antimicrobial resistance (AMR) panel to monitor AMR, which has been periodically expanded. The panel systematically monitors resistance to substances including amoxicillin, ampicillin, cefotaxime, gentamicin, neomycin, doxycycline, and colistin, providing essential data on the growing global challenge of AMR [1,2].

*Enterococcus* species are Gram-positive bacteria commonly found in the gastrointestinal tracts of animals and humans, as well as in natural bodies of water and food products [3,4]. These catalase-negative, non-spore-forming, facultative anaerobic lactic acid-producing bacteria [5] have become one of the leading causes of nosocomial infections globally, driven by increasing antimicrobial resistance over the past 50 years [6,7]. These opportunistic pathogens frequently cause severe and resistant infections in humans, such as endocarditis, bacteremia, and urinary tract infections [5,8].

The pathogenic potential of the genus was first detailed by MacCallum and Hastings in a patient with endocarditis, establishing the commensal–opportunistic paradigm of the genus [4]. However, until 1984, *Enterococcus* species were classified under the genus *Streptococcus* [9,10]. The genus *Enterococcus* now comprises more than 50 species, among which *E. faecalis* and *E. faecium* are the most common inhabitants of both human and animal gut microbiota [4]. By the 1990s, *E. faecium* accounted for 90–95% of human clinical isolates. However, with the spread of antimicrobial resistance, the proportion of *E. faecalis* isolates began to rise, particularly due to the dissemination of vancomycin and ampicillin resistance [11,12]. *E. faecalis* and *E. faecium* account for 80% of human hospital-acquired *Enterococcus* infections [13], and, after *Staphylococcus* species, infections caused by *Enterococcus* species are the leading cause of nosocomial infections, with an associated mortality rate of 23% in human healthcare settings [14].

The gastrointestinal tracts of animals serve as the largest reservoir for *Enterococcus* species [12], with transmission between animals and humans occurring bidirectionally [15]. Consequently, the strict implementation of and compliance with biosecurity measures are crucial for preventing disease transmission and ensuring effective outbreak control [16].

Antibiotic use exerts selective pressure, contributing to AMR through horizontal gene transfer between animals and humans [17,18]. The misuse of antibiotics accelerates resistance development across multiple drug classes, including aminoglycosides, β-lactams, macrolides, tetracyclines, and streptogramins [19]. Consequently, global efforts have been focused on replacing antibiotics either partially or entirely with alternative strategies. The main use of antibiotics we can replace is preventive use, but promising therapeutic alternatives are also currently under development [20]. As a response to AMR, alternative approaches such as antimicrobial peptides [21], medium-chain fatty acids [22], various plant-based essential oils and extracts [23,24,25,26], and propolis [27,28,29] have been developed. These alternatives are increasingly explored to reduce antibiotic dependency, particularly in high-usage sectors like swine [30] and poultry farming [31]. Additionally, optimizing antibiotic selection through pharmacokinetic/pharmacodynamic evaluations may further mitigate selective pressure [32].

Pathogenic *Enterococcus cecorum* strains are of growing concern due to their rapid transmission via inhalation and oro-fecal routes [33,34]. Despite this bacterium being a normal intestinal microbiota component in poultry, its pathogenic strains can spread within flocks, yet their exact vertical transmission mechanisms remain unclear [35]. Further research is needed to determine how these strains breach the intestinal barrier and whether co-infections contribute to systemic disease [33,34,36].

Several food-borne pathogens, such as *Enterococcus* species, are able to enter the food chain and may reach a wide range of consumers through various meat products. Their mere presence and multiplication would itself be a significant problem, but they are also capable of transferring various resistance genes. This represents a major concern for food chain safety, which requires particular attention during the production and processing of certain foods, such as turkey, which is an increasingly popular source of protein in the diets of many people. In 2021, the global turkey meat market reached an annual production volume of 6.2 million tons, a 12.7% increase since 2007 [37]. Projections estimate that production will rise to 6.7 million tons by 2025 [37]. The United States leads turkey meat production with 2.7 million tons (43% of global production); Mexico (157,000 tons) and Germany (117,000 tons) have emerged as the largest importers [38] due to specific regional factors. In Mexico, domestic turkey production is insufficient to meet the high consumer demand, leading to substantial imports to bridge this gap. In Germany, the popularity of turkey meat has risen, driven by health-conscious consumers seeking lean protein alternatives, resulting in increased imports to satisfy this growing preference [39].

In Hungary, 7.5–8.5 million turkeys are raised annually, resulting in approximately 90,000 tons of raw cuts, frozen meat, and further-processed products, with 65,000–70,000 tons of turkey products produced. Domestic demand accounts for 30,000–35,000 tons, with the surplus destined for export [40]. In Hungary, the turkey stock at the end of 2022 was 2,519,000 birds [41]. Hungary has a notable meat production sector, particularly in poultry and pork. However, compared to larger European producers like Germany, Spain, and France, Hungary’s total meat production volume is relatively modest. This difference can be attributed to factors such as the size of the agricultural sector, available infrastructure, and domestic demand. Hungarian consumers exhibit unique preferences in meat consumption. A study analyzing ultra-processed food consumption across Europe found that in Hungary, the intake of ultra-processed dishes (UPDs) was higher than that of ultra-processed foods (UPFs), a trend also observed in Belgium, Denmark, the Netherlands, Portugal, and Romania. This suggests a cultural inclination toward traditional or minimally processed meat dishes [42,43]. Consumer attitudes toward meat products vary across Europe. In Hungary, organic production methods are considered less important compared to in other countries, while the region of origin of products holds significant value. Additionally, the Hungarian retail sector shows a high proportion of domestic food products, with 70.85% of analyzed food items supplied by domestic companies and an even higher proportion observed in fresh food categories [42,44].

The EFSA has identified *E. faecalis* as the most significant AMR reservoir bacterium in poultry within the European Union [45]. Several studies have confirmed that *E. faecalis* strains adapted to human hospitals originate from poultry, further reinforcing the connection between animal and human health under the One Health framework [46].

Given the economic importance of turkey production and the emerging role of *Enterococcus* species in infections of public health relevance, we aimed to conduct a comprehensive susceptibility study of *Enterococcus* strains isolated from turkeys in Hungary.

## 2. Results

### 2.1. Regional Distribution and Origin of Samples

**Figure 1:**
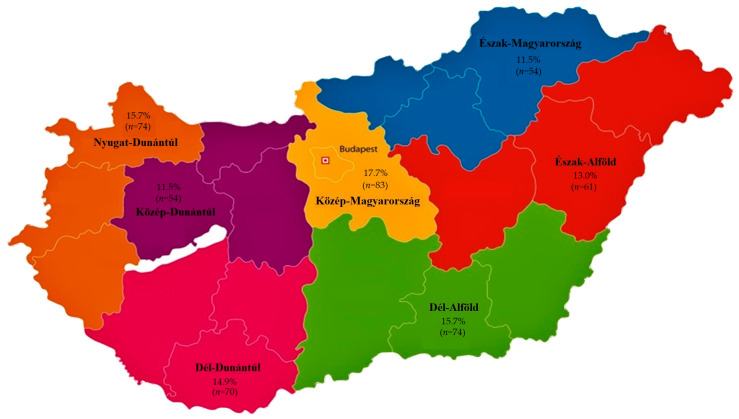
The regional distribution of *Enterococcus* strains (*n* = 470) isolated from turkeys across the seven administrative regions of Hungary.

A total of 470 *Enterococcus* isolates from turkeys were subjected to susceptibility testing against 15 antibiotics of significance in both veterinary and public health contexts. The samples were collected from 21 livestock farms across Hungary, ensuring nationwide coverage with representation from at least 3 farms in each region to achieve a nearly representative sampling between 2022 and 2023. From each farm, 15 oral–pharyngeal swab samples and 15 cloacal swab samples were obtained, resulting in a total of 315 animals sampled. Both types of swabs were collected from the same animals by the attending veterinarians and sent to the reference laboratory for isolation. Regarding sample distribution, 12.9% originated from Észak-Alföld, 15.7% from Dél-Alföld, 17.7% from Közép-Magyarország, 15.7% from Nyugat-Dunántúl, 14.9% from Dél-Dunántúl, 11.5% from Észak-Magyarország, and 11.5% from Közép-Dunántúl regions. Of these samples, 47.4% were respiratory and 52.6% were cloacal swabs (Figure 1). Additionally, 70.6% of the samples came from meat-producing flocks, while 29.4% originated from breeding farms. The majority of the samples (86.4%) were collected from smaller farms (5001–50,000 birds), while 13.6% were from larger farms (50,001–100,000 birds).

### 2.2. Antimicrobial Susceptibility Testing

**Figure 2:**
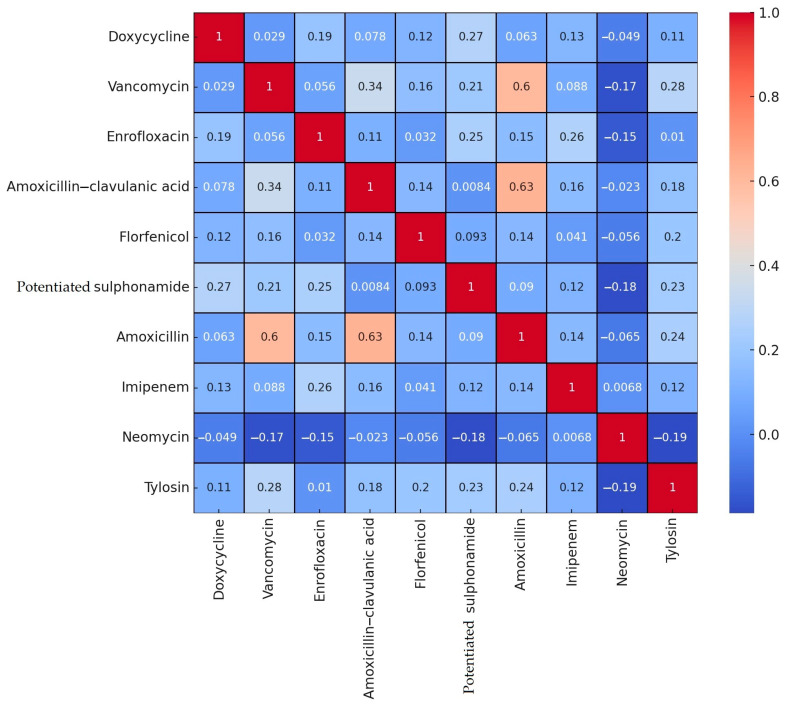
Correlation heatmap of the degree of resistance of *Enterococcus* samples (*n* = 499) isolated from turkeys to certain drugs. The heatmap shows the correlation coefficients as color blocks, where red shades indicate positive correlation and blue shades indicate negative correlation. The stronger the color, the stronger the correlation.

A total of 10 antibiotics were included for analysis, for which Clinical Laboratory Standard Institute (CLSI) breakpoints or data from the literature were available. The resistance levels determined for these antibiotics were subjected to correlation analysis and visualized using a heatmap (Figure 2).

A relatively strong positive correlation (0.63) was observed between amoxicillin and amoxicillin–clavulanic acid, suggesting that resistance to one often implies resistance to the other. Similarly, a positive correlation (0.60) was detected between amoxicillin and vancomycin. In contrast, the most notable negative correlations were observed between neomycin and tylosin (−0.19), neomycin and potentiated sulfonamide (−0.18), and neomycin and vancomycin (−0.17).

**Figure 3:**
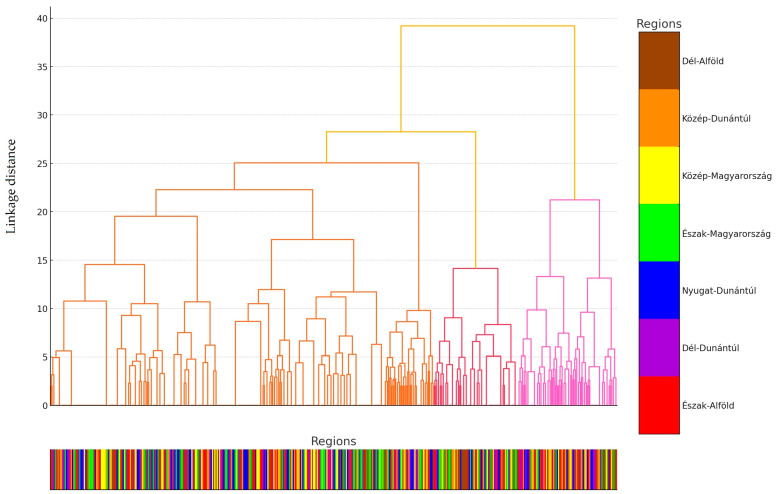
Cluster analysis is followed by a strain-by-strain plot of the dendrogram generated according to the antimicrobial resistance profile based on the distance of each cluster. The different colors help to visually separate the clusters.

Individual samples were grouped into distinct clusters based on their resistance patterns. The proximity of clusters indicates similar resistance profiles among the samples. The Y-axis represents the distance, reflecting the degree of difference between the samples. Smaller distances indicate greater similarity in resistance patterns, with samples located at lower distances (toward the bottom) being highly similar, while clusters at higher distances (toward the top) are more distinct (Figure 3).

**Figure 4:**
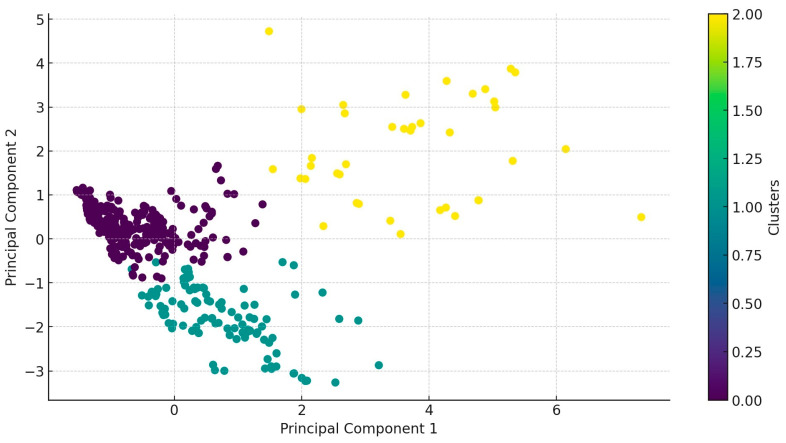
After principal component analysis, the data are grouped into three clusters. Each color represents a different cluster rank into which samples are classified (purple—0; green—1; yellow—2).

We identified three clusters: Cluster 0 (272 samples), Cluster 1 (153 samples), and Cluster 2 (45 samples). In Cluster 0, sample distribution across regions was relatively even, except for a significantly lower sample count from the Dél-Dunántúl region. In Cluster 1, the sample count was notably high in the Közép-Magyarország region, while no samples were collected from the Észak-Magyarország region. In Cluster 2, the Dél-Alföld and Dél-Dunántúl regions showed significantly higher sample counts. The characteristics of the clusters demonstrated significant differences in antibiotic resistance patterns. The clusters are well separated on the principal component analysis (PCA) plot (Figure 4), and the dendrogram further clarifies the hierarchical structure. The regional distribution of clusters suggests potential geographic variation in the spread of antibiotic resistance.

When resistance patterns were examined for individual antibiotics, doxycycline, potential sulfonamide, enrofloxacin, and imipenem were clustered closely together (in purple), indicating similarities in resistance patterns. Florfenicol and tylosin grouped into another cluster (in green), showing similar resistance patterns, while a third cluster included amoxicillin, amoxicillin–clavulanic acid, and vancomycin (in yellow). Antibiotics positioned near each other on the plot exhibit similar resistance patterns. This visualization aids in understanding resistance patterns and optimizing treatment strategies.

We conducted a statistical analysis of resistance levels for each antibiotic based on the sample source (respiratory or cloacal), utilization type (meat or breeding), age group (juvenile or adult), and flock size (5001–50,000 or 50,001–100,000) (Table 1). The sample source had the least influence, with significant differences observed only in resistance to doxycycline and imipenem. Utilization type and age group were equally influential, and in fact identical, with significant differences in all antibiotics except doxycycline, enrofloxacin, and potentiated sulfonamide. Flock size had less impact overall.

For these comparisons, an independent *t*-test was used. The *t*-test assumes a normal distribution of the data; where this assumption was not met, the Mann–Whitney U-test was applied. While antibiotic resistance data may not always meet the strict assumption of normality, the *t*-test is considered robust against minor deviations. Our null hypothesis (H_0_) for all comparisons was that there is no difference in the mean resistance rates between the two groups. The alternative hypothesis (H_1_) posited that a difference in mean resistance rates does exist between the groups.

**Figure 5:**
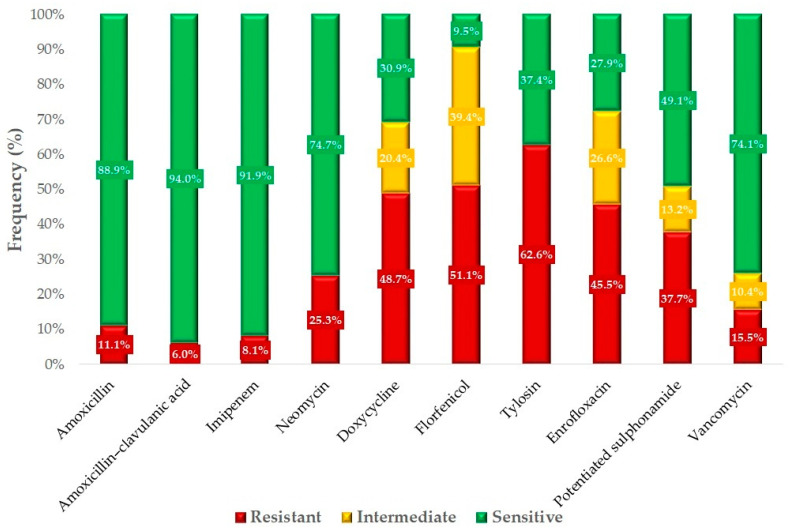
Antimicrobial resistance profile of *Enterococcus* strains isolated from turkeys (*n* = 470).

Based on the available breakpoints, we determined the resistance profiles of the isolates for 10 antibiotics (Figure 5). The majority of isolates retained sensitivity to amoxicillin, which is a first-choice veterinary treatment option for infections caused by these strains, with a resistance rate of 11.1%. However, significant resistance was observed against tylosin (62.6%), florfenicol (51.1%), doxycycline (48.7%), and enrofloxacin (45.5%). The 15.5% resistance to vancomycin is particularly concerning.

We created frequency tables for the minimum inhibitory concentration (MIC) values obtained for each antibiotic (Table 2) and calculated MIC_50_ and MIC_90_ values. Where epidemiological cut-off values (ECOFFs) defined by the European Committee on Antimicrobial Susceptibility Testing (EUCAST) were available, these were also included. MIC_50_ represents the antibiotic concentration that inhibits the growth of 50% of the tested bacterial population, while MIC_90_ indicates the concentration that inhibits 90%. Both MIC_50_ and MIC_90_ values remained below the breakpoint for imipenem and amoxicillin–clavulanic acid. The MIC_50_ value stayed below the breakpoint for neomycin, doxycycline, vancomycin, amoxicillin, enrofloxacin, and potentiated sulfonamide. Among the tested antibiotics, the MIC_50_ value was below the ECOFF threshold for imipenem and vancomycin.

The ECOFF is an MIC value distinguishing wild-type (non-resistant) microorganisms from those exhibiting resistance mechanisms. It identifies microorganisms that have likely acquired resistance mechanisms, making it a critical tool in tracking the spread of antibiotic resistance. The ECOFF sets the threshold below which antibiotic-sensitive wild-type populations are found. Microorganisms with values above this threshold are likely to possess resistance mechanisms.

For antibiotics without defined breakpoints or those not practical for routine use, the frequency tables are provided in Supplementary Table S1.

We were able to propose tentative ECOFFs for antimicrobials exhibiting a bimodal MIC distribution (Supplementary Table S2).

For amoxicillin, a tentative value of 8 µg/mL was determined (Supplementary Figure S1), suggesting that at least half of the examined population can be classified as wild type. Similarly, a tentative ECOFF of 16 µg/mL was identified for amoxicillin–clavulanic acid (Supplementary Figure S2), indicating that at least 90% of the population could be considered wild type.

For imipenem, a tentative ECOFF of 32 µg/mL was observed (Supplementary Figure S3), which substantially exceeds the EUCAST reference value of 4 µg/mL. In the case of florfenicol, a tentative value of 16 µg/mL was determined (Supplementary Figure S4), exceeding the 8 µg/mL EUCAST reference, suggesting that at least half of the tested population falls within the wild-type category.

A considerably higher tentative value of 64 µg/mL was observed for enrofloxacin (Supplementary Figure S5), implying that at least half of the examined population may belong to the wild-type group. Finally, for vancomycin, the tentative ECOFF was 4 µg/mL (Supplementary Figure S6), which aligns with the EUCAST-defined reference, suggesting that at least half of the tested isolates can be considered wild type.

**Figure 6:**
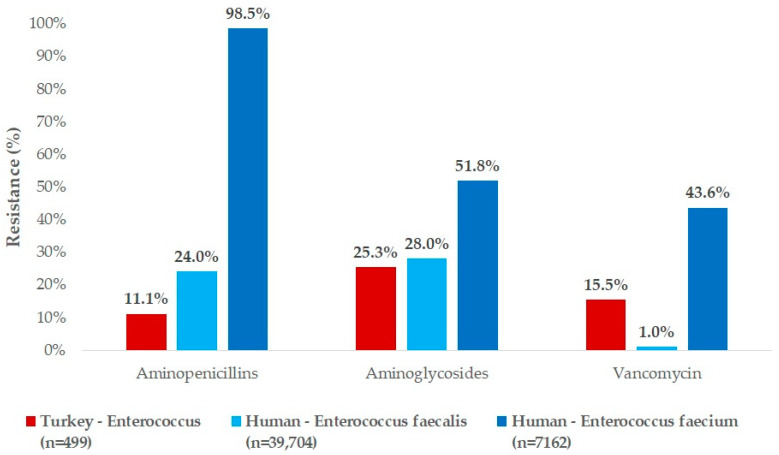
Sensitivity profile of human and turkey *Enterococcus* strains isolated from turkeys, based on a comparison of available active substances.

We compared our findings with human resistance data from clinical cases in Hungary (Figure 6). There were notable differences in resistance levels. For aminopenicillins, the resistance rate to amoxycillin in turkey-derived strains was significantly lower than the resistance to ampicillin observed in human strains. For aminoglycosides (neomycin), the resistance level in turkey-derived strains was very similar to the resistance observed for gentamicin in human *E. faecalis* strains, but half that of *E. faecalis* strains. Resistance to vancomycin in turkey-derived strains was notably higher than the 1% resistance observed in human *E. faecalis* strains but much lower than the 43.6% resistance found in human *E. faecium* strains.

## 3. Discussion

In our study, we determined the susceptibility profiles of 470 turkey-derived commensal *Enterococcus* strains to 10 antimicrobial agents of veterinary and public health importance. Nationwide, 11.1% resistance to amoxicillin was observed. Comparatively, Woźniak-Biel et al. reported 17% resistance in *E. faecalis* and 66.7% in *E. faecium* strains [47], while Kempf et al. found no resistance (0%) [48]; Makarov et al. reported 5.9% resistance to ampicillin [49]. Our findings indicate that amoxicillin has retained its efficacy against *Enterococcus* infections, which is significant given its status as a first-line antibiotic. Although several international studies have reported considerably higher resistance rates, these differences are likely due to varying patterns of antibiotic use. Notably, ampicillin holds greater importance in human medicine, and cross-resistance may occur among aminopenicillins.

For amoxicillin–clavulanic acid, a resistance rate of 6% was observed, closely aligning with the resistance rate for amoxicillin. This consistency supports the understanding that *Enterococcus* species do not produce β-lactamase enzymes and so are unaffected by clavulanic acid. However, no comparative research is available, as clavulanic acid does not have a defined maximum residue limit (MRL) for poultry.

For vancomycin, a resistance rate of 15.5% was identified. Similarly, Woźniak-Biel et al. reported resistance rates of 14.6% in *E. faecalis* and 22.2% in *E. faecium* strains [47], while Makarov et al. [49] and Boulianne et al. found no resistance [50]. Vancomycin remains a life-saving agent in human medicine for treating multidrug-resistant *Enterococcus* infections. The resistance rate observed in our study is within the expected range but underscores the necessity of regular monitoring for this critical antibiotic. As we examined commensal strains, their role as reservoirs for antimicrobial resistance is particularly significant.

Among fluoroquinolones, enrofloxacin exhibited a resistance rate of 45.5%. In contrast, Woźniak-Biel et al. reported ciprofloxacin resistance rates of 12.2% for *E. faecalis* and 77.8% for *E. faecium* strains [47], while Makarov et al. observed 100% resistance [49], and Boulianne et al. identified a resistance rate of 12.2% [50]. Fluoroquinolones are critically important antibiotics, whose use must be significantly reduced. Furthermore, they are not the primary agents of choice for treating *Enterococcus* infections, due to their classification as critically important antibiotics for human health. However, their public health importance, particularly in hospital settings, underscores the necessity of continuous monitoring studies.

Among aminoglycosides, we observed a resistance rate of 25.3% for neomycin. In comparison, Makarov et al. reported a significantly higher resistance rate of 94.1% [49], while Boulianne et al. recorded a resistance rate of 33.8% [50]. Among tetracyclines, resistance to doxycycline was 48.7%, whereas Woźniak-Biel et al. reported resistance rates of 92.2% for *E. faecalis* strains and 100% for *E. faecium* strains [47]. Similarly, both Makarov et al. [49] and Boulianne et al. [50] observed 100% resistance to tetracyclines. Aminoglycosides and tetracyclines have been overused for several decades, resulting in generally high resistance levels. However, certain hospital-acquired infections treated with aminoglycosides like gentamicin are a reminder of their critical importance.

For tylosin, we observed a resistance rate of 62.6%, whereas Boulianne et al. reported a significantly higher resistance rate of 94.2% [50]. Regarding imipenem, our study identified a resistance rate of 8.1%. There is no comparative research specifically addressing turkeys. However, Roy et al. reported a much higher resistance rate of 55.6% in broiler chickens [51], while Schwaiger et al. found comparable rates in laying hens, with 4.3% resistance under organic keeping and 5.5% under conventional keeping systems [52]. Despite the variation in these findings, it is important to remember that carbapenems are “last-resort” antibiotics, reserved for critical use in human healthcare, and therefore, any resistance to them is entirely undesirable.

For florfenicol, we recorded a resistance rate of 51.5%. Though comparative data for turkeys are currently unavailable, Schwaiger et al. did not detect any resistant strains in laying flocks of chickens [52], while Osman et al. reported a significantly higher resistance rate of 83.3% in chickens [53].

For potentiated sulfonamides, we observed a resistance rate of 37.7%, whereas Kempf et al. reported resistance rates ranging between 16.7% and 33.3% [48]. The widespread use of sulfonamides over several decades has contributed to the significant prevalence of resistance, leading to reduced usage in recent years. Periodic reduction in the use of certain antibiotics could support the re-emergence of susceptible strains, potentially restoring their effectiveness over time.

Antibiotics showing strong positive correlations are less effective when used in combination, as resistance to one often extends to the other. The correlation matrix of antibiotics can guide the selection of optimal antibiotic combinations, reducing the risk of resistance. These findings provide valuable insights into resistance patterns and facilitate more targeted preventive measures.

In this study, tentative ECOFFs were determined based on MIC distributions using ECOFFinder. By applying a 99% threshold, we ensured that the defined cut-off encompassed the vast majority of the wild-type population, minimizing the risk of misclassification. For certain antimicrobials, the tentative ECOFFs identified in our study were higher than the EUCAST reference values.

For imipenem, the tentative ECOFF was 32 µg/mL, which substantially exceeds the EUCAST reference value of 4 µg/mL. Similarly, for florfenicol, a tentative ECOFF of 16 µg/mL was determined, exceeding the EUCAST value of 8 µg/mL. This suggests that the MIC distribution of wild-type strains in the examined population may differ from previously published data. The tentative ECOFF for enrofloxacin was 64 µg/mL, considerably higher than the EUCAST reference, indicating that the MIC distribution in the studied population may vary geographically. For amoxicillin, a tentative ECOFF of 8 µg/mL was determined, while for amoxicillin–clavulanic acid, the value was 16 µg/mL, suggesting that at least 90% of the examined population can be classified as wild-type.

The results obtained with ECOFFinder exhibited a good fit to the observed MIC distributions, as confirmed by the symmetrical distribution of residuals. This indicates that the method may be suitable for estimating tentative ECOFFs in cases where EUCAST reference values are unavailable [54,55,56]. However, while these tentative values can aid in distinguishing between wild-type and non-wild-type strains, they should not be considered final, standardized ECOFFs. The findings do not replace the official ECOFFs defined by EUCAST, but rather serve as complementary estimates, particularly in populations where MIC distributions deviate significantly from those reported in previous studies. Further research and larger-scale investigations are necessary to validate and refine the tentative ECOFFs determined in this study.

The study revealed that the source of the sample, whether from the trachea or cloaca, had a minimal influence on resistance levels. This is likely related to the behavior of poultry, which frequently peck at the ground, potentially leading to the colonization of the oral–pharyngeal mucosa by the same strains present in fecally contaminated environments. The significance of utilization types was similarly demonstrated in our previous investigations with chickens [57]. In turkeys, age groups correlate with longer rearing periods and differing antibiotic usage patterns. Farm size appears to have a lesser impact on resistance differences. The underlying cause may be the presence of similar selective pressures.

When human resistance data were compared with those from turkeys, the proportion of resistant strains to amoxicillin was significantly lower in turkeys than in human samples. A comprehensive human study reported a median resistance rate of 81.8% for ampicillin among *E. faecium* strains [58], while Zacharopoulos et al. documented a decline in resistance from 21.8% during 2010–2014 to 0.7% during 2017–2021 [59]. For neomycin, resistance in turkeys was 25.3%, which closely aligned with the 28.0% resistance observed for gentamicin in human *E. faecalis* strains. Billström et al. reported 2% resistance among *E. faecium* strains [60], while Taji et al. found 50.9% resistance in *E. faecalis* strains [61]. For vancomycin, resistance in turkey isolates was 15.5%, compared to 1% in human *E. faecalis* and 43.6% in human *E. faecium* isolates. A comprehensive human study reported a median vancomycin resistance rate of 11.0% for *E. faecium* strains [58]. Additionally, an EU-wide human study documented vancomycin resistance rates of 14.9% in 2017, 17.3% in 2018, and 18.3% in 2019 [62], showing a steadily increasing trend.

A limitation of comparing turkey and human isolates is the differing sample sizes and the lack of species-specific separation in the turkey isolates. Nonetheless, our findings underscore the need for regular monitoring studies of this kind. Commensal strains are established reservoirs of resistance, and correlating results from commensal strains, clinical isolates, and human data forms a critical foundation for the One Health approach.

## 4. Materials and Methods

### 4.1. The Origin of Samples and Human Data

The examined strains were collected between 2022 and 2023 during routine diagnostic investigations conducted by veterinarians serving large-scale livestock farms in Hungary. The selection of farms was entirely random, with the only criterion being that samples were collected from at least three farms per administrative region. The animals selected for sampling were chosen entirely at random. For each sample, data were recorded regarding the organ of origin (trachea or cloaca), the location of the farm, the type of flock (meat, egg, or breeding), the age group (juvenile or adult), and the size of the flock (5001–50,000; 50,001–100,000; >100,001).

The samples were collected by practicing veterinarians using sterile Amies-type swabs without charcoal and equipped with standard aluminum shafts (Biolab Zrt., Budapest, Hungary). For each animal, two swabs were taken: one oral–pharyngeal sample near the tracheal entrance and one cloacal sample. The swabbing procedure involved rotating the swab in a circular motion 3–5 times. A total of 315 animals were sampled, yielding 630 swab samples. From these, 470 *Enterococcus* strains were successfully isolated.

After collection, the samples in transport media were shipped to the reference laboratory under temperature-controlled conditions (2–8 °C). Upon arrival, the samples were plated on m-*Enterococcus* modified agar (Merck KGaA, Darmstadt, Germany), following the manufacturer’s instructions. Only *Enterococcus* species grow on this medium, and the pure cultures from the samples were stored at −80 °C using the Microbank™ system (Pro-Lab Diagnostics, Richmond Hill, ON, Canada).

Human resistance data were provided by the Hungarian National Public Health and Pharmaceutical Center. The data, including resistance percentage values, both aggregated and region-specific, were provided with approval from the National Chief Medical Officer.

### 4.2. Minimum Inhibitory Concentration (MIC) Determination

Phenotypic resistance expression was assessed by determining the MIC values, following the methodology outlined by the CLSI [63]. Breakpoints were also established according to CLSI guidelines [64] and compared with the ECOFF defined by EUCAST. For neomycin, a breakpoint of 1024 µg/mL was identified from a study examining meat products for *Enterococcus* species [65]. Similarly, a breakpoint of 8 µg/mL was found for tylosin, based on literature [66].

The bacterial strains, stored at −80 °C, were suspended in 3 mL of cation-adjusted Müller–Hinton broth (CAMHB) the day before testing and incubated at 37 °C for 18–24 h. The tests were conducted using 96-well microtiter plates (VWR International, LLC, Debrecen, Hungary). Except for the first column, all wells were filled with 90 µL of CAMHB. Stock solutions of the tested antibiotics (Merck KGaA, Darmstadt, Germany) at a concentration of 1024 µg/mL were prepared according to CLSI guidelines [64].

The active ingredients amoxicillin and amoxicillin–clavulanic acid in a 2:1 ratio (pH 7.2, 0.01 mol/L) and imipenem (pH 6, 0.1 mol/L) were dissolved in phosphate buffer solution. Doxycycline, neomycin, tylosin, and vancomycin were dissolved in distilled water. For the preparation of potentiated sulfonamide (trimethoprim and sulfamethoxazole at a 1:19 ratio), sulfamethoxazole was dissolved in hot water with a few drops of 2.5 mol/L NaOH, while trimethoprim was dissolved in distilled water with 0.05 mol/L HCl. Enrofloxacin was prepared using a few drops of 1 mol/L NaOH solution in distilled water. Florfenicol was dissolved using a few drops of 95% ethanol and distilled water. A two-fold serial dilution series was created, beginning with 180 µL of the stock solution diluted to 512 µg/mL in CAMHB and distributed across the wells. Excess liquid (90 µL) was discarded after the 10th column, leaving 90 µL in each well.

A bacterial suspension adjusted to the 0.5 McFarland standard using a nephelometer (ThermoFisher Scientific, Budapest, Hungary) was inoculated into the microtiter plates from the 11th column backward at 10 µL per well [63]. MIC evaluation was performed using the Sensititre™ SWIN™ automatic MIC reader (ThermoFisher Scientific, Budapest, Hungary) and the VIZION system software version 3.4 (ThermoFisher Scientific, Budapest, Hungary, 2024). The quality control strain used was *E. faecalis* (ATCC 29212).

### 4.3. The Determination of Epidemiological Cut-Off Values

ECOFFs were determined using ECOFFinder version 2.1. [67]. A 99% threshold was selected, representing the MIC value that encompasses 99% of the modeled wild-type population, thereby increasing specificity in defining wild-type isolates.

For this analysis, MIC frequency distributions were generated within the tested antimicrobial concentration range (1024–0.001 µg/mL). Data entry required specifying the bacterial species and the antibiotic tested, and indicating whether the dataset was derived from more than one MIC distribution. Additionally, the total number of observations had to be provided.

During analysis, a Nest starting value was required, which had to be close to but not equal to the total number of isolates. The Solver macro in the ECOFFinder template was then executed to identify the subset of data points that provided the best fit to the observed values. The program subsequently calculated residuals, defined as the differences between observed values and the fitted curve estimates. The selected subset was considered reliable if the residuals were symmetrically distributed on both sides of the zero reference line.

Finally, the software generated a graphical representation illustrating how well the fitted values aligned with the observed MIC data.

### 4.4. Statistical Analyses

Statistical analysis was conducted using R program version 4.1.0 [68]. The normality of data distribution was assessed with the Shapiro–Wilk test (shapiro.test function in the stats package), and non-parametric tests were applied to data that did not follow a normal distribution. The Kruskal–Wallis test (kruskal.test function in the stats package) was used to evaluate resistance levels to different antibiotics across various categories [69]. This test does not assume normality and is suitable for comparing medians across multiple groups, making it ideal for analyzing differences among categories. Subsequently, post hoc tests, including Mann–Whitney U tests (wilcox.test function in the stats package) [70] and *t*-tests (t.test function in the stats package), were performed to determine specific group relationships. Pairwise comparisons for all types were conducted, and *p*-values were corrected for inflation due to multiple comparisons using the Bonferroni correction method (p.adjust function in the stats package) [71]. It is important to note that Bonferroni correction can increase the risk of Type II errors (failing to detect true differences). Further analyses included correlation studies to examine relationships between antibiotics and principal component analysis (PCA) [72], which helped identify similarities or differences among samples (prcomp function in the stats package). Hierarchical cluster analysis (hclust function in the stats package) was also performed, visualized with a dendrogram [73]. This figure represented the distances between samples and the hierarchical structure of clustering.

The correlation (cor.test function in the stats package) coefficient (ranging from −1 to 1) indicates the extent to which two variables (in this case, resistance to two antibiotics) vary together. A value of 1 signifies perfect positive correlation, meaning if a sample is resistant to one antibiotic, it is certainly resistant to the other. A value of 0 indicates no correlation, implying that resistance to one antibiotic does not affect resistance to the other. A value of −1 signifies perfect negative correlation, indicating that if a sample is resistant to one antibiotic, it is certainly not resistant to the other.

## 5. Conclusions

This study examined the resistance levels of *Enterococcus* in turkeys and compared these levels with those found in samples from human cases. Overall, there appears to be much lower resistance in turkeys than in humans, indicating that differing strains may be present and that resistance in different species may be developing independently.

Our findings indicate that amoxicillin, a penicillin commonly used to treat *Enterococcus* infections, has retained its efficacy, with low resistance levels observed in turkeys. However, data from human cases reveal significant differences in the sensitivity profiles of the two dominant species, *E. faecium* and *E. faecalis*. This highlights the importance of identifying bacterial strains in future studies and the need to investigate the genetic basis of resistance in multidrug-resistant strains using next-generation sequencing.

From a public health perspective, the observed 15.5% vancomycin resistance aligns closely with international trends. As vancomycin-resistant strains are often treated with linezolid in human healthcare as a last-resort antibiotic, future studies should also assess sensitivity to this agent.

Our results shed light on the antimicrobial resistance status of commensal *Enterococcus* strains in Hungarian turkey flocks. However, the literature has shown that AMR develops over time, and so the need for periodic monitoring to establish trends cannot be over-emphasized. Comparisons with public health data reinforce the One Health concept and encourage collaborative efforts. In the future, we aim to integrate antibiotic usage data from individual farms into our analysis, which may reveal further correlations and insights into resistance dynamics.

## Figures and Tables

**Table 1:** Statistical analysis of resistance by sampling (respiratory, cloacal), utilization (meat, breeding), age group (growing, adult), and herd size (5001–50,000; 50,001–100,000).

Antibiotics	Respiratory–Cloacal	Meat–Breeding	^3^ Young–^4^ Adult	^5^ Small–^6^ Medium
	*p*-Values			
Doxycycline	0.0020 *	0.2439	0.2439	0.7508
Vancomycin	0.2667	<0.0001 *	<0.0001 *	0.0031 *
Enrofloxacin	0.4012	0.2107	0.2107	<0.0001 *
^1^ Amoxicillin–clavulanic acid	0.6169	0.0019 *	0.0019 *	0.1105
Florfenicol	0.6949	0.0063 *	0.0063 *	0.5336
^2^ Potentiated sulfonamide	0.1269	0.1423	0.1423	<0.0001 *
Amoxicillin	0.6966	<0.0001 *	<0.0001 *	0.0294 *
Imipenem	0.0887 *	0.0109 *	0.0109 *	0.0106 *
Neomycin	0.4533	<0.0001 *	<0.0001 *	<0.0001 *
Tylosin	0.1241	0.0001 *	0.0001 *	0.2714

* Significant difference (*p* < 0.05); ^1^ trimethoprim–sulfamethoxazole 1:19 ratio; ^2^ 1:2 ratio; ^3^ younger than 6 weeks; ^4^ older than 6 weeks; ^5^ small—5001–50,000 birds; ^6^ medium—50,001–100,000 birds.

**Table 2:** Frequency table of the minimum inhibitory concentration (MIC) values of active substances with breakpoints obtained from *Enterococcus* of turkey-origin samples (*n* = 470). The top row of each active substance shows the number of pieces, and the bottom row shows the percentage of each. The red vertical line indicates the breakpoints.

Antibiotic	^1^ BP *	0.001	0.002	0.004	0.008	0.016	0.031	0.063	0.125	0.25	0.5	1	2	4	8	16	32	64	128	256	512	1024	MIC_50_	MIC_90_	^2^ ECOFF	^3^ T
	µg/mL																						µg/mL			
Enrofloxacin	^1^ 4				1	7	15	3	2	21	82	91	34	61	47	31	24	27	11	8	3	2	2	64	-	64
					0.2%	1.5%	3.2%	0.6%	0.4%	4.5%	17.4%	19.4%	7.2%	13.0%	10.0%	6.6%	5.1%	5.7%	2.3%	1.7%	0.6%	0.4%				
^4^ Potentiated sulfonamide	^1^ 4								1	3	20	44	38	53	72	42	20	8	19	13	59	78	1	16	-	-
									0.2%	0.6%	4.3%	9.4%	8.1%	11.3%	15.3%	8.9%	4.3%	1.7%	4.0%	2.8%	12.6%	16.6%				
Tylosin	8						1	0	2	2	4	71	72	24	13	7	1	3	9	26	74	161	256	1024	-	-
							0.2%	0.0%	0.4%	0.4%	0.9%	15.1%	15.3%	5.1%	2.8%	1.5%	0.2%	0.6%	1.9%	5.5%	15.7%	34.3%				
Florfenicol	^1^ 8										2	6	37	185	141	46	12	24	16	0	1		8	32	8	16
											0.4%	1.3%	7.9%	39.4%	30.0%	9.8%	2.6%	5.1%	3.4%	0.0%	0.2%					
Doxycycline	^1^ 16					1	2	0	17	9	2	37	25	52	96	126	64	39					8	32	1	-
						0.2%	0.4%	0.0%	3.6%	1.9%	0.4%	7.9%	5.3%	11.1%	20.4%	26.8%	13.6%	8.3%								
Amoxicillin	^1^ 16	1	0	0	1	3	4	4	20	49	96	132	59	38	11	4	6	3	6	12	10	11	1	32	-	8
		0.2%	0.0%	0.0%	0.2%	0.6%	0.9%	0.9%	4.3%	10.4%	20.4%	28.1%	12.6%	8.1%	2.3%	0.9%	1.3%	0.6%	1.3%	2.6%	2.1%	2.3%				
Imipenem	^1^ 16	15	0	1	9	7	6	8	6	20	78	126	85	40	31	24	3	1	6	1	2	1	1	8	4	32
		3.2%	0.0%	0.2%	1.9%	1.5%	1.3%	1.7%	1.3%	4.3%	16.6%	26.8%	18.1%	8.5%	6.6%	5.1%	0.6%	0.2%	1.3%	0.2%	0.4%	0.2%				
^5^ Amoxicillin–clavulanic acid	^1^ 16	2	0	0	1	3	5	3	14	43	102	110	92	53	14	10	12	5	1				1	4	-	16
		0.4%	0.0%	0.0%	0.2%	0.6%	1.1%	0.6%	3.0%	9.1%	21.7%	23.4%	19.6%	11.3%	3.0%	2.1%	2.6%	1.1%	0.2%							
Vancomycin	^1^ 32						1	0	5	8	20	130	183	1	43	6	5	2	7	29	12	18	2	256	4	4
							0.2%	0.0%	1.1%	1.7%	4.3%	27.7%	38.9%	0.2%	9.1%	1.3%	1.1%	0.4%	1.5%	6.2%	2.6%	3.8%				
Neomycin	1024									2	1	6	10	8	17	10	26	62	68	67	74	119	256	1024	256	-
										0.4%	0.2%	1.3%	2.1%	1.7%	3.6%	2.1%	5.5%	13.2%	14.5%	14.3%	15.7%	25.3%				

* BP—breakpoint; ^1^ Clinical Laboratory Standard Institute (CLSI); ^2^ epidemiological cut-off value (EUCAST); ^3^ our tentative ECOFFs; ^4^ trimetophrime–sulfamethoxazole 1:19 ratio; ^5^ 2:1 ratio.

## Data Availability

The data presented in this study are available from the corresponding author upon reasonable request.

## Supplementary Materials

The following supporting information can be downloaded at https://www.mdpi.com/article/10.3390/antibiotics14040331/s1. Table S1: Frequency table of the minimum inhibitory concentration (MIC) values (µg/mL) for agents without breakpoints in *Enterococcus* samples derived from turkeys (*n* = 470). The top row for each agent shows the count, while the bottom row shows the percentage. Table S2: Tentative epidemiological cut-off values (ECOFFs) calculated from the MIC distributions of *Enterococcus* strains (*n* = 470) isolated from turkeys with a bimodal distribution. Figure S1: MIC distribution of *Enterococcus* strains (*n* = 470) isolated from turkeys for amoxicillin. Figure S2: MIC distribution of *Enterococcus* strains (*n* = 470) isolated from turkeys for amoxicillin–clavulanic acid. Figure S3: MIC distribution of *Enterococcus* strains (*n* = 470) isolated from turkeys for imipenem. Figure S4: MIC distribution of *Enterococcus* strains (*n* = 470) isolated from turkeys for florfenicol. Figure S5: MIC distribution of *Enterococcus* strains (*n* = 470) isolated from turkeys for enrofloxacin. Figure S6: MIC distribution of *Enterococcus* strains (*n* = 470) isolated from turkeys for vancomycin.
