# Supplementary material for: Antimicrobial Susceptibility Profiles of Commensal Enterococcus spp. Isolates from Turkeys in Hungarian Poultry Farms Between 2022 and 2023
Source: Antibiotics (Basel). 2025 Mar 21;14(4):331. doi: 10.3390/antibiotics14040331 (PMC12024081; doi:10.3390/antibiotics14040331)
Supplement: Supplementary file 1 [file antibiotics-14-00331-s001.zip › antibiotics-3507536-supplementary.pdf]

Supplementary Table S1 Frequency table of the minimum inhibitory concentration (MIC) values (µg/mL) for agents without breakpoints in *Enterococcus* samples derived from turkeys (*n*=470). The top row for each agent shows the count, while the bottom row shows the percentage.

Antibiotic	0.001	0.002	0.004	0.008	0.016	0.03	0.06	0.125	0.25	0.5	1	2	4	8	16	32	64	128	256	512	1024	MIC_{50}	MIC_{90}
																						µg/mL	
Ceftriaxone						1	9	4	9	8	14	10	10	13	18	14	11	48	51	102	148	512	1024
						0.2%	1.9%	0.9%	1.9%	1.7%	3.0%	2.1%	2.1%	2.8%	3.8%	3.0%	2.3%	10.2%	10.9%	21.7%	31.5%		
Spectinomycin												1	0	1	13	38	96	120	88	31	82	128	1024
												0.2%	0.0%	0.2%	2.8%	8.1%	20.4%	25.5%	18.7%	6.6%	17.4%		
Tiamulin									1	4	10	1	0	4	4	9	21	103	140	120	53	256	1024
									0.2%	0.9%	2.1%	0.2%	0.0%	0.9%	0.9%	1.9%	4.5%	21.9%	29.8%	25.5%	11.3%		
Lincomycin									1	1	7	1	5	10	22	33	80	56	34	98	122	256	1024
									0.2%	0.2%	1.5%	0.2%	1.1%	2.1%	4.7%	7.0%	17.0%	11.9%	7.2%	20.9%	26.0%		
Colistin														27	7	3	8	7	15	113	290	1024	1024
														5.7%	1.5%	0.6%	1.7%	1.5%	3.2%	24.0%	61.7%		

Supplementary Table S2 Tentative epidemiological cutoff values (ECOFFs) calculated from the MIC distributions of *Enterococcus* strains (*n*=470) isolated from turkeys with a bimodal distribution.

**Agent**	**Species**	**Distributions**	**Total Observations**	**Selected Subset**	**Modal MIC**	**Log2MIC Mode**	**Max Log2MIC**	**Selected Log2 Mean**	**Selected Log2 SD**	**CV**	**ECOFF 97.5% Exact**	**ECOFF 97.5% R'd-up**	**ECOFF 99 % Exact**	**ECOFF 99% R'd-up**	**Dilution Range**	**% @ ECOFF 97.5**	**% @ ECOFF 99**	**Date analysed**
Amoxicillin	*Enterococcus spp.*	21	470	≤ 8	1	0	10	-0.80448	1.43412	1.29855	4.01759	8	5.78279	8	8	0.02127	0.02127	2025.03.17
Amoxicillin clavulanic acid	*Enterococcus spp.*	21	470	≤ 16	1	0	7	-0.51059	1.57135	1.50829	5.93476	8	8.84526	16	9	0.04231	0.01069	2025.03.17
Imipenem	*Enterococcus spp.*	21	470	≤ 64	1	0	10	-0.34608	1.97348	2.34434	11.4865	16	18.9603	32	10	0.03116	0.01045	2025.03.17
Florfenicol	*Enterococcus spp.*	21	470	≤ 16	4	2	9	1.918015	0.79365	0.59449	11.1082	16	13.5886	16	4	0.08204	0.08204	2025.03.17
Enrofloxacin	*Enterococcus spp.*	21	470	≤ 32	1	0	10	0.23719	2.39579	3.84237	30.5449	32	56.1276	64	11	0.03474	0.01533	2025.03.17
Vancomycin	*Enterococcus spp.*	21	470	≤ 4	2	1	10	0.029587	0.64769	0.47254	2.46063	4	2.90056	4	4	0.06586	0.06586	2025.03.17

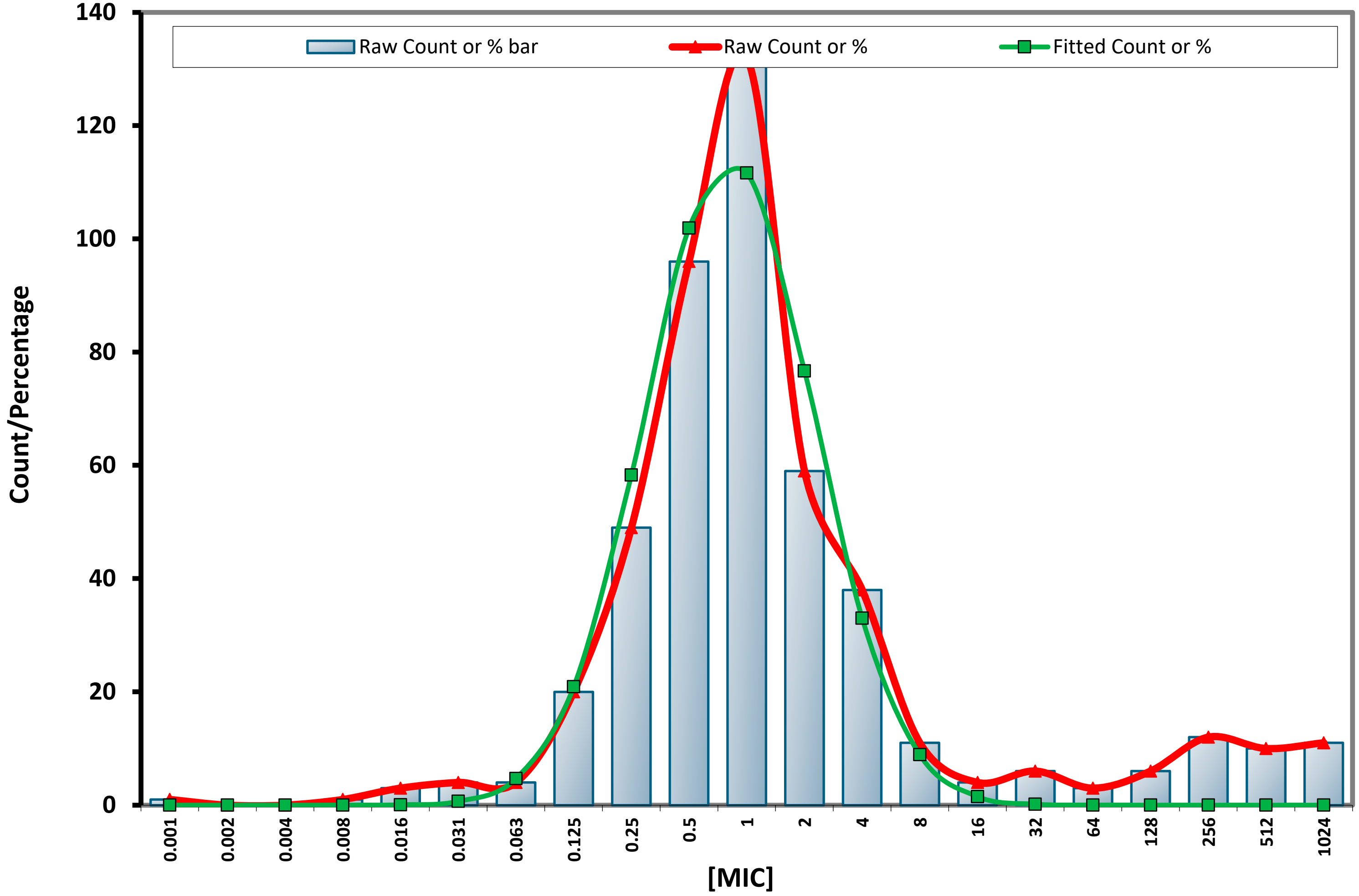

Supplementary Figure S1 MIC distribution of *Enterococcus* strains (*n*=470) isolated from turkeys for amoxicillin. The raw count (red line) represents the observed MIC distribution, while the fitted count (green line) represents the modeled density distribution. The tentative epidemiological cutoff value (ECOFF) for amoxicillin was estimated at 8 µg/mL, distinguishing the wild-type population from non-wild-type isolates.

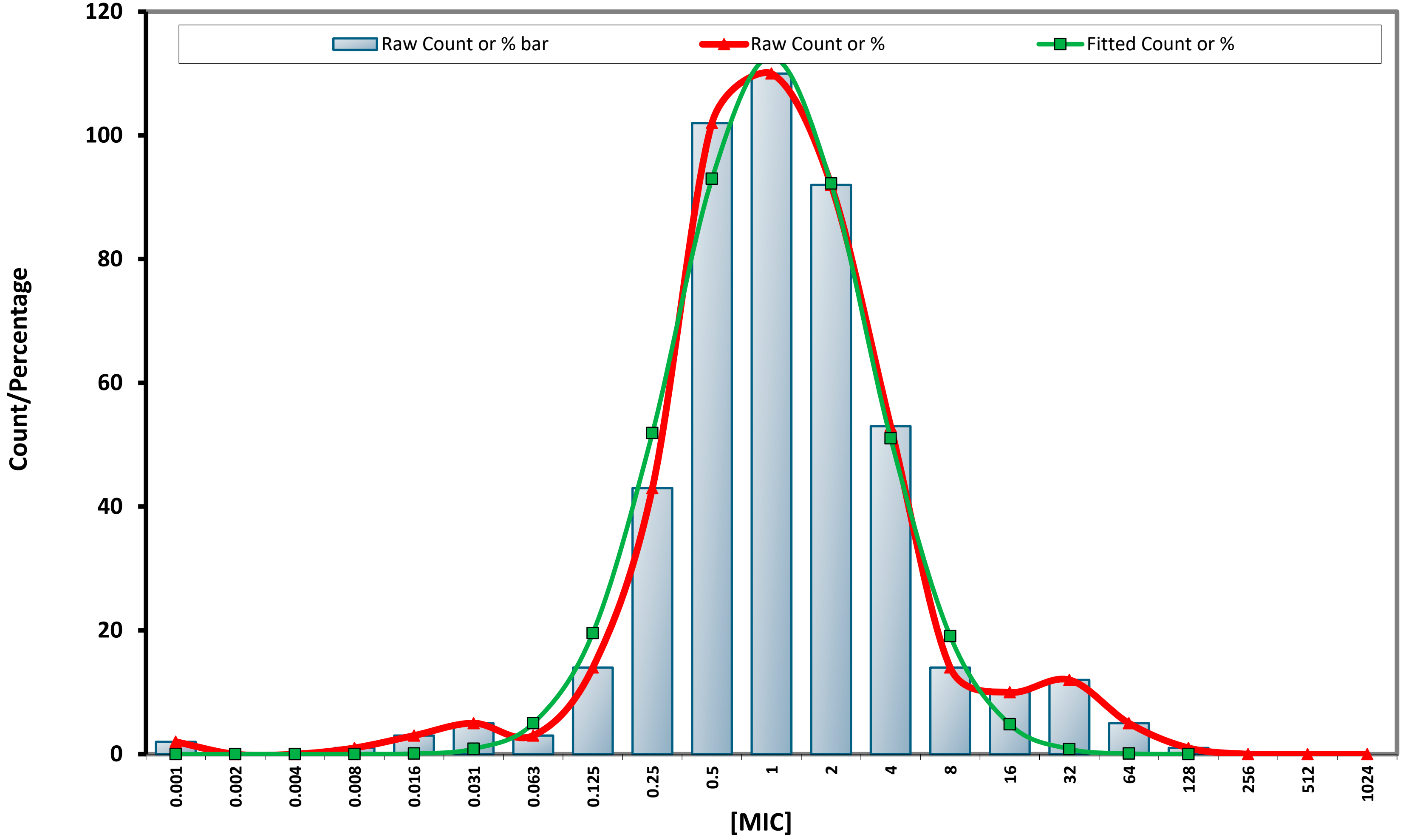

Supplementary Figure S2 MIC distribution of *Enterococcus* strains (*n*=470) isolated from turkeys for amoxicillin clavulanic acid. The raw count (red line) represents the observed MIC distribution, while the fitted count (green line) represents the modeled density distribution. The tentative epidemiological cutoff value (ECOFF) for amoxicillin clavulanic acid was estimated at 16 μg/mL, distinguishing the wild-type population from non-wild-type isolates.

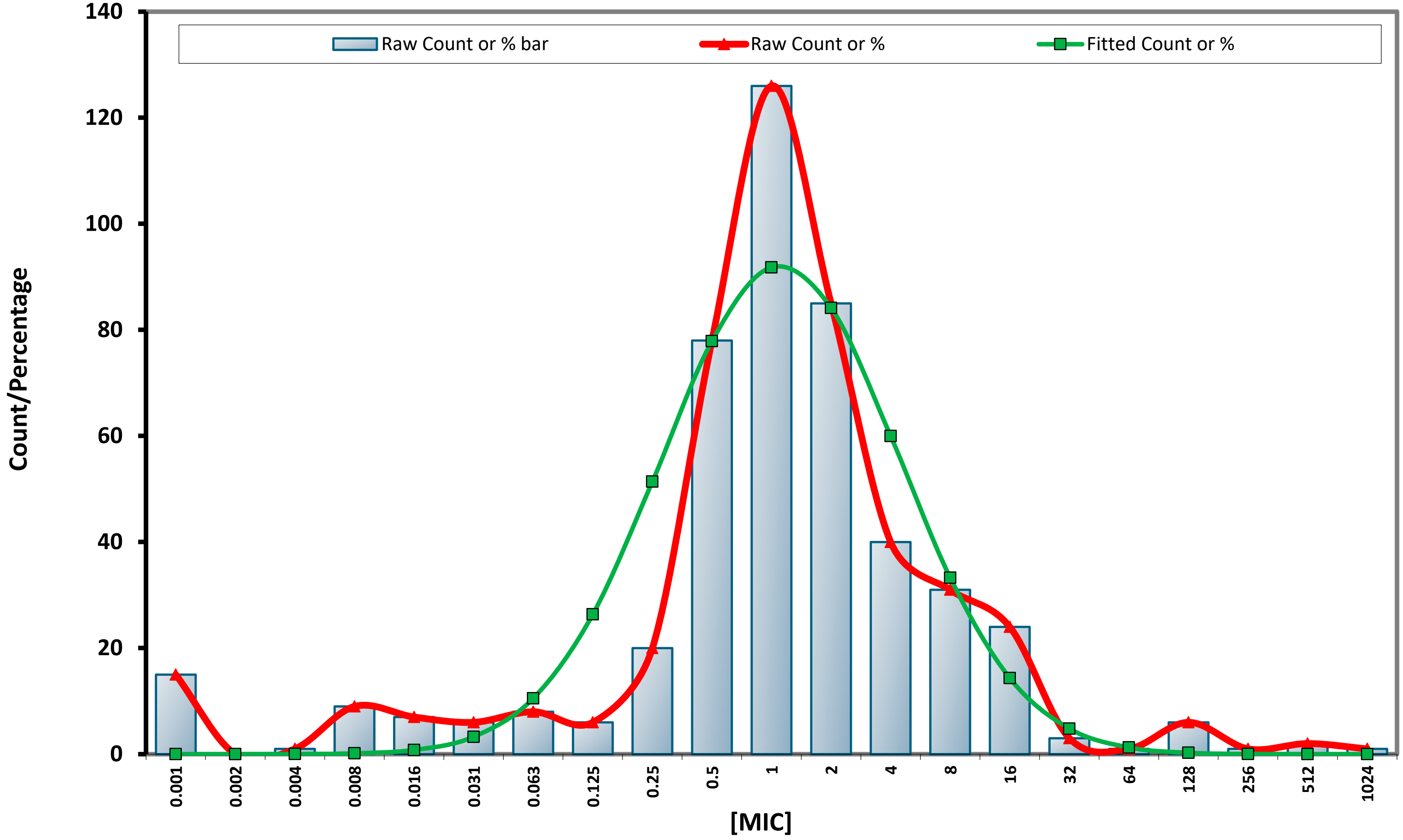

Supplementary Figure S3 MIC distribution of *Enterococcus* strains (*n*=470) isolated from turkeys for imipenem. The raw count (red line) represents the observed MIC distribution, while the fitted count (green line) represents the modeled density distribution. The tentative epidemiological cutoff value (ECOFF) for imipenem was estimated at 32 μg/mL, distinguishing the wild-type population from non-wild-type isolates.

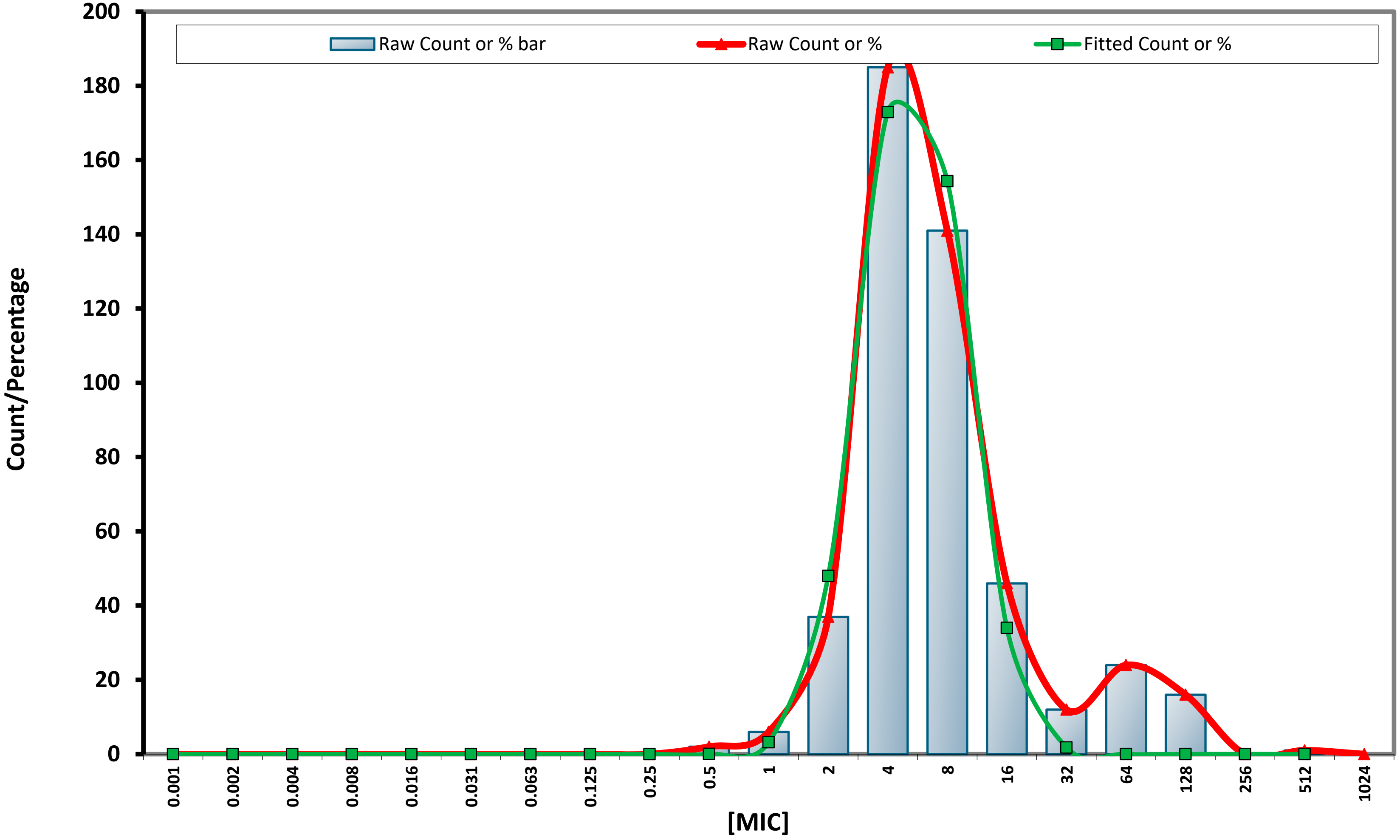

Supplementary Figure S4 MIC distribution of *Enterococcus* strains (*n*=470) isolated from turkeys for florfenicol. The raw count (red line) represents the observed MIC distribution, while the fitted count (green line) represents the modeled density distribution. The tentative epidemiological cutoff value (ECOFF) for florfenicol was estimated at 16 μg/mL, distinguishing the wild-type population from non-wild-type isolates.

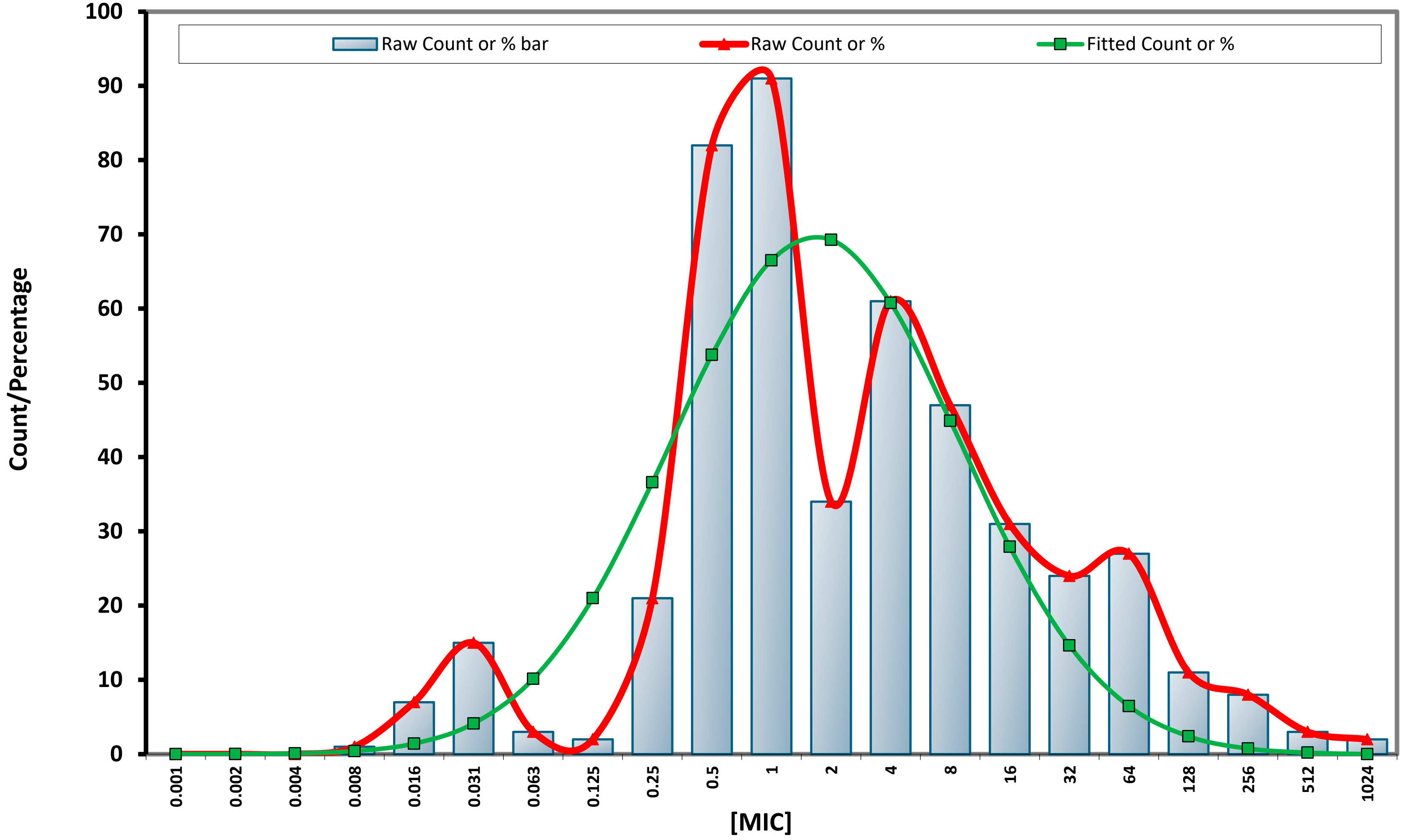

Supplementary Figure S5 MIC distribution of *Enterococcus* strains (*n*=470) isolated from turkeys for enrofloxacin. The raw count (red line) represents the observed MIC distribution, while the fitted count (green line) represents the modeled density distribution. The tentative epidemiological cutoff value (ECOFF) for enrofloxacin was estimated at 64 µg/mL, distinguishing the wild-type population from non-wild-type isolates.

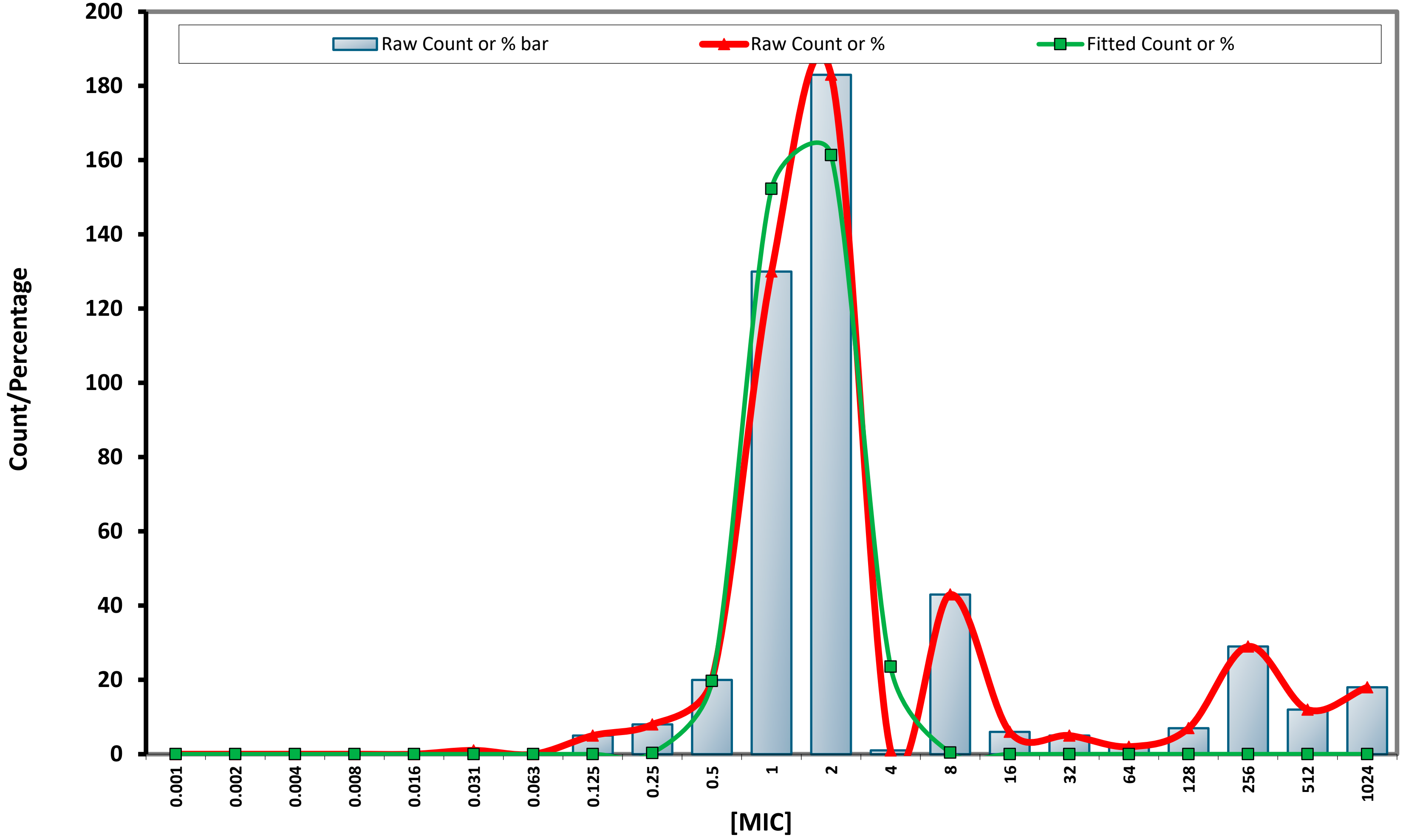

Supplementary Figure S 6 MIC distribution of *Enterococcus* strains (*n*=470) isolated from turkeys for vancomycin. The raw count (red line) represents the observed MIC distribution, while the fitted count (green line) represents the modeled density distribution. The tentative epidemiological cutoff value (ECOFF) for vancomycin was estimated at 4 µg/mL, distinguishing the wild-type population from non-wild-type isolates.
